# Applying switchable Cas9 variants to in vivo gene editing for therapeutic applications

**DOI:** 10.1007/s10565-019-09488-2

**Published:** 2019-08-15

**Authors:** Emily M. Mills, Victoria L. Barlow, Louis Y. P. Luk, Yu-Hsuan Tsai

**Affiliations:** School of Chemistry, Cardiff University, Cardiff, CF10 3AT UK

**Keywords:** Endonuclease, Gene therapy, In vivo gene editing, Switchable Cas9

## Abstract

Progress in targeted gene editing by programmable endonucleases has paved the way for their use in gene therapy. Particularly, Cas9 is an endonuclease with high activity and flexibility, rendering it an attractive option for therapeutic applications in clinical settings. Many disease-causing mutations could potentially be corrected by this versatile new technology. In addition, recently developed switchable Cas9 variants, whose activity can be controlled by an external stimulus, provide an extra level of spatiotemporal control on gene editing and are particularly desirable for certain applications. Here, we discuss the considerations and difficulties for implementing Cas9 to in vivo gene therapy. We put particular emphasis on how switchable Cas9 variants may resolve some of these barriers and advance gene therapy in the clinical setting.

Gene therapy is a tool to treat or cure diseases by modifying a patient’s genotype. Modifications include replacing a lack-of-function gene with its wild-type sequence, silencing a disease-causing gene that is constitutively active, or introducing a new gene of novel function to treat a disease (Dangi et al. 2018; Gupta and Shukla 2017). Therefore, gene therapy is complementary to existing therapeutic approaches and provides an obvious solution to diseases with a clear genetic origin (Anguela and High 2019). Importantly, gene therapy is perhaps the only permanent and inheritable solution for diseases caused by a lack-of-function gene (Cox et al. 2015). Due to its unique advantages, there has been significant research on its use to tackle otherwise hard-to-treat diseases, invoking a surge of gene therapies entering clinical trials (Naldini 2015). Although most clinical trials rely on ex vivo gene editing, in which cells are removed from the patient, modified, and then transferred back, in recent years, more trials are being conducted to explore in vivo gene editing in animal models (Naldini 2015). The in vivo approach is particularly beneficial for diseases of the muscles and internal organs, such as Duchenne muscular dystrophy, cystic fibrosis, and tyrosinemia (Cox et al. 2015; Wang et al. 2017; Mention et al. 2019).

This review limits the discussion to in vivo gene editing for therapeutic applications. The premise of all gene editing techniques involves breaking a double-stranded DNA under the action of an endonuclease (Fig. 1a), followed by repair via cellular machinery (Fig. 1b). The mechanism of repair can result in a few different outcomes. For example, the double-strand break can be repaired into a new, defined sequence to correct a diseased gene, or be disrupted to inactivate a disease-causing gene with autosomal dominant effects. In comparison to other gene editing systems, the clustered regularly interspaced short palindromic repeats (CRISPR) system is unique. In particular, CRISPR associated protein 9 (Cas9) is specifically appealing due to its high activity and flexibility in experimental design (Wang et al. 2017; Kim and Kim 2014). Moreover, recent advances in protein engineering have yielded switchable Cas9 variants, whose activity can be controlled with spatiotemporal resolution by an external stimulus (Gangopadhyay et al. 2019; Nihongaki et al. 2018; Richter et al. 2017; Zhou and Deiters 2016), and we envisage that such variants developed by us (Suzuki et al. 2018) and others (Zetsche et al. 2015; Davis et al. 2015; Oakes et al. 2016; Liu et al. 2016; Nguyen et al. 2016; Tang et al. 2017; Senturk et al. 2017; Rose et al. 2017; Nihongaki et al. 2015; Hemphill et al. 2015; Richter et al. 2016; Jain et al. 2016; Zhou et al. 2018) can benefit clinical development of in vivo gene editing.

**Fig. 1:**
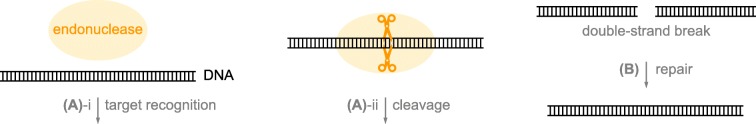
Two stages of precise gene editing involving **a** recognition of the target DNA by the endonuclease and subsequent cleavage to generate a double-strand break and **b** repair of the break by cellular mechanisms

In this review, we will first illustrate the molecular mechanisms of gene editing and provide examples of Cas9-mediated gene editing in disease models. We will then compare different means to deliver Cas9 for in vivo gene therapy. Finally, we will discuss how switchable Cas9 variants may advance gene therapy in a clinical setting*.*

## Cas9 for gene editing

Endonucleases that generate a double-strand break at the targeted DNA sequence are indispensable for gene editing (Cox et al. 2015; Cornu et al. 2017). There have been many endonucleases discovered to date that can cause site-specific double-strand breaks, and many have been developed for the purpose of gene therapy. Such endonucleases include meganucleases, zinc-finger nucleases, transcriptional activator-like effector nucleases, and CRISPR/Cas9 (Cox et al. 2015). Among them, Cas9, found in the Gram-positive bacterium *Streptococcus pyogenes* (*Sp*Cas9), is arguably the most versatile (Wang et al. 2017). Although its use in precise gene editing was initially questioned by its relatively high off-target activity, this issue has been progressively addressed through different approaches (Wang et al. 2017), including engineering of *Sp*Cas9 variants with negligible non-specific activity (Slaymaker et al. 2016; Kleinstiver et al. 2016; Hu et al. 2018).

**Fig. 2:**
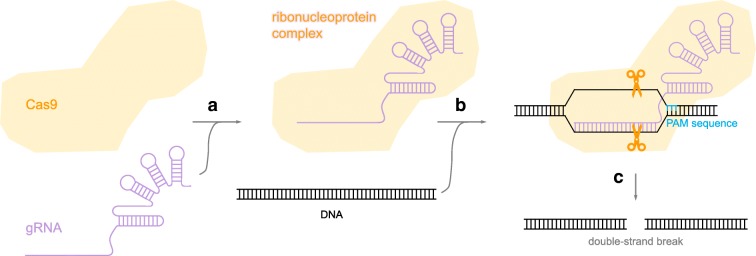
Generation of a DNA double-strand break by Cas9 involving **a** formation of the ribonucleoprotein, **b** recognition of the target DNA, and **c** cleavage of the double-stranded DNA

The popularity of *Sp*Cas9 originates from its flexibility in creating DNA double-strand breaks at different target sequences (Cox et al. 2015). The specificity of other endonucleases relies on the amino acid sequence of the DNA-binding domain. Consequently, when a new DNA target is required, researchers must alter and engineer the DNA-binding domain of the endonuclease to achieve specificity to the new target. Clearly, this process can be labor-intensive and time-consuming, if not challenging. In stark contrast, the specificity of *Sp*Cas9 can be determined by a single-strand guide RNA (gRNA) molecule, rather than the protein domain within the enzyme (Jinek et al. 2012). Accordingly, DNA double-strand breaks at desired sites can be easily introduced by the addition of corresponding gRNA molecules, which are readily achievable using standard cloning techniques. Specifically, a gRNA molecule contains two parts: (i) a 20-nucleotide guide sequence that complements the DNA target and (ii) a structural motif essential for binding the enzyme *Sp*Cas9. At the molecular level (Jiang and Doudna 2017), a ribonucleoprotein (a complex of *Sp*Cas9 and gRNA) is first formed (Fig. 2a). In order for the complex to bind to the target DNA (Fig. 2b), the existence of a protospacer adjacent motif (PAM) upstream of the DNA target sequence in the genome is essential (Jinek et al. 2012). Guided by the PAM sequence and complementarity to the DNA target, the gRNA in the ribonucleoprotein forms a double-stranded complex with DNA. Consequently, the target DNA is cleaved by *Sp*Cas9 of the ribonucleoprotein (Fig. 2c), generating a double-strand break (Jiang and Doudna 2017). For wild-type *Sp*Cas9, 5′-NGG-3′ is the required PAM sequence, but *Sp*Cas9 variants that recognize different PAM sequences have also been generated (Wang et al. 2017; Hu et al. 2018). Therefore, it is theoretically possible to use *Sp*Cas9 and its variants to target nearly any gene. Given the simplicity and versatility, there has been an exponential use of *Sp*Cas9 in in vitro and in vivo genome editing reported in the literature (Dangi et al. 2018; Nishitani et al. 2019).

## Cellular DNA repair mechanisms

**Fig. 3:**
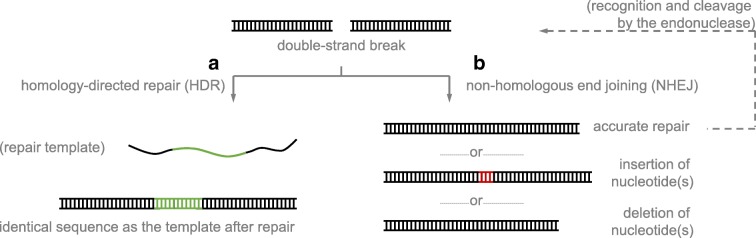
Consequences of repairing a double-strand break by the two cellular mechanisms, **a** homology-directed repair (HDR) and **b** non-homologous end joining (NHEJ)

When a double-strand DNA break is formed inside a cell, there are two major repair mechanisms: homology-directed repair (HDR, Fig. 3a) and non-homologous end joining (NHEJ, Fig. 3b) (Wyman and Kanaar 2006). In HDR, a DNA molecule with identical nucleotide sequence (i.e., homology) to the two sides of the break is present and used by the cell as the template to repair the lesion accordingly. This ensures the repaired DNA molecule will have identical nucleotide sequence to the template. The DNA repair template can be of endogenous or exogenous origin, and an exogenously supplied template can be either single- or double-stranded DNA (Pawelczak et al. 2018). In the case of double-stranded DNA, it can be a linear fragment or a circular plasmid (Pawelczak et al. 2018). For gene editing, it is also possible to supply a template containing a new gene of novel function to the cell. On the other hand, during repair by NHEJ, the two DNA fragments are reconnected without a template. In this mechanism, accurate repair yielding an identical sequence to that before the cleavage is the most prominent outcome (Fig. 3b), although indels (insertion or deletion of nucleotides) can also be produced at the cleavage site. However, if the double-strand break is generated by an endonuclease, products of accurate repair retain the recognition sequence and are readily re-cleaved by the endonuclease, whereas indel products are not. Therefore, in the absence of a repair template, indel products will accumulate and become the predominant consequence over time. Since indels in exon sequences often lead to a frameshift with a premature stop codon, the open reading frame of the gene is disrupted. Subsequently, mRNA resulting from such a disrupted gene is either recognized and degraded by the nonsense-mediated decay pathway, or translated into truncated, non-functional protein. The overall outcome is gene silencing, and this mechanism has been commonly used for gene knockout (Wyman and Kanaar 2006; Pawelczak et al. 2018).

Generally, in mammalian systems, most DNA repairs undergo the NHEJ pathway which is active at all stages of the cell division cycle, whereas the HDR mechanism is less efficient and restricted to the S and G_2_ phases (Wyman and Kanaar 2006; Pawelczak et al. 2018). Nevertheless, HDR is desired in many scenarios of gene editing therapy (see below). HDR efficiency can be increased by controlling the activity of the endonuclease at the G_2_ and S phases, administration of small molecules (e.g., cell cycle arrest drugs, or inhibitors of proteins involved in the NHEJ pathway), or optimization of repair DNA template format (Pawelczak et al. 2018; Robert et al. 2015; Paulk et al. 2012). However, it is still technically challenging to achieve close to 100% HDR efficiency in mammalian models even when taking these approaches.

## Diseases benefitting from in vivo gene editing

As HDR products always have the correct sequence restored, this can theoretically be used to restore any genetic disorder. Indeed, most therapeutic gene editing research thus far relies on HDR to correct diseased mutations (Cox et al. 2015; Wang et al. 2017; Cornu et al. 2017; Nishitani et al. 2019; Yin et al. 2014a). For example, HDR is particularly suitable to correct in-frame nonsense mutations, or multiple clustered mutations simultaneously. However, the relative inefficiency of HDR limits its applications to diseases where low-efficiency editing can still significantly improve gene function and disease pathology. For example, a small percentage of gene correction to exhibit functional restoration is beneficial to diseases, such as cystic fibrosis (Schwank et al. 2013; Hodges and Conlon 2019), Duchenne muscular dystrophy (Duan 2015; Long et al. 2014, 2016; Bogdanovich et al. 2002), Huntington’s disease (Kolli et al. 2017; Carroll et al. 2011; Kordasiewicz Holly et al. 2012), retinal dysfunction (Min et al. 2005; Narfström et al. 2003), severe combined immunodeficiency (Gaspar et al. 2004), and tyrosinemia (Yin et al. 2014a, 2016).

The NHEJ mechanism could provide an efficient therapeutic option for single-gene autosomal dominant disorders. Such disorders are caused by mutations of a sole gene on one of the autosomal (i.e. non-sex) chromosomes. Huntington’s disease (Glorioso et al. 2015) and epidermolysis bullosa simplex (Lewin et al. 2005) are two such examples. Because patients of these diseases retain one copy of the wild-type gene, knockout of the mutated version via NHEJ will infer the recessive wild-type copy to regain normal protein function. This general concept of silencing the single disease-causing allele has been proven to be effective in a number of knockdown studies by antisense oligonucleotides (Seyhan 2011). Although the use of Cas9 to silence a gene has not been fully explored (Kolli et al. 2017; Christie et al. 2017; Bakondi et al. 2016), it is theoretically possible to use Cas9-mediated knockout to replace antisense oligonucleotides for gene silencing.

Alternatively, DNA repair by the NHEJ approach can be used to restore a gene silenced by certain indels. One-nucleotide insertion is the most prevalent outcome (> 20%) of NHEJ (Cradick et al. 2013; Sürün et al. 2018; Chen et al. 2018a), so this repair mechanism can restore the open reading frame of genes containing specific indels (i.e., …, − 4, − 1, 2, 5, …). Indeed, this concept has been proven in cellular models of X-linked chronic granulomatosis disease, where the production of full-length cytochrome b-245 heavy chain increased by 25% as the result of non-templated NHEJ repair (Sürün et al. 2018).

**Fig. 4:**
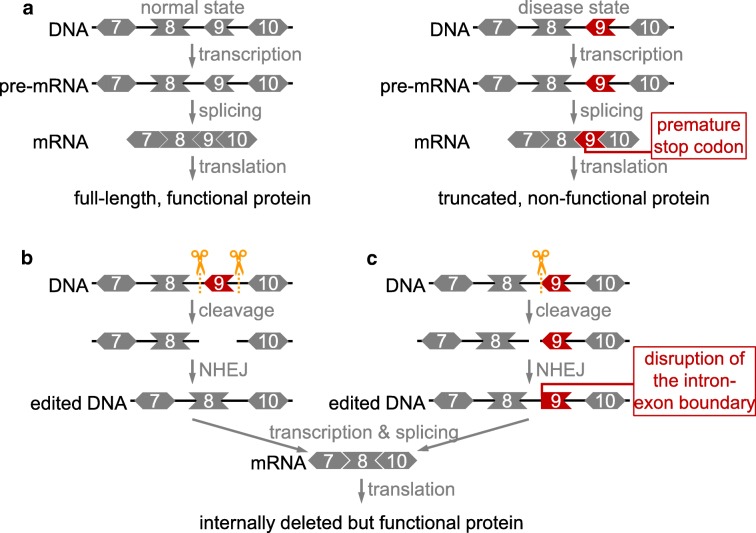
Exon skipping by NHEJ to restore an open reading frame. **a** Protein production from a normal or disease state DNA. **b** Exon skipping by cutting out the mutant exon. **c** Exon skipping by disrupting the intron-exon boundary

NHEJ can also be used to produce a functional protein by exon skipping, and this approach has been explored as a new gene therapy in animal models (Long et al. 2016; Chen et al. 2018b; Amoasii et al. 2018; Xu et al. 2016; Aartsma-Rus et al. 2017; Turczynski et al. 2016; Touznik et al. 2014). Many mammalian genes contain several exons that together code for the amino acid sequence in the final protein product (Fig. 4a). If a disease-causing mutation or frame shift occurs in a region not critical for protein function, exon skipping can be used to produce a shorter, but functional, protein. This can be achieved by using two gRNA molecules to direct Cas9 to cut each end of the mutant exon, followed by non-templated NHEJ to connect the adjacent DNA, resulting in removal of the mutant exon (Fig. 4b). Alternatively, exon skipping can be achieved by disrupting the intron-exon junction (Fig. 4c). Both strategies have been successfully demonstrated in mouse models of Duchenne muscular dystrophy (Long et al. 2016; Chen et al. 2018b; Amoasii et al. 2018; Xu et al. 2016). Furthermore, exon skipping approaches have already been investigated in other genetic diseases, including muscular dystrophy (Aartsma-Rus et al. 2017), dystrophic epidermolysis bullosa (Turczynski et al. 2016), and neuromuscular diseases (Touznik et al. 2014). Therefore, it is foreseeable that development of Cas9-mediated exon skipping will be of clinical interest.

Gene editing can involve exploitation of either of the endogenous cellular repair pathways, and both have been studied in vivo for genetic mutation correction (Table 1). A key advantage of HDR is that the entire section of DNA can be replaced with a corrected sequence, restoring the gene to its wild-type sequence. This contrasts with NHEJ which produces indels that may restore the open reading frame, induce exon skipping, or lead to gene knockout. While both may result in resolution of disease phenotype, only HDR can result in a DNA sequence indistinguishable from the wild-type sequence. For both repair mechanisms, prediction tools are available to analyze any target gene. For HDR, the most promising gRNA sequences for high-efficiency Cas9 editing can be generated (O’Brien et al. 2018). The nature of indels resulting from NHEJ can be predicted using tools which analyze the most likely outcome for a particular nucleotide sequence (Chen et al. 2018a). These tools can be used with target gene sequences to select suitable splice sites that will increase the probability of a preferred indel, and therefore the desired outcome. However, as repair via NHEJ is more efficient than that of HDR (Wyman and Kanaar 2006; Pawelczak et al. 2018), it would be beneficial to employ NHEJ whenever possible.

**Table 1:** Examples of in vivo genetic engineering to treat genetic diseases

Disease	Gene	Form of delivery	Repair	Reference
Tyrosinemia type 1	*Fah*	Hydrodynamic injection	HDR	(Yin et al. 2016)
Tyrosinemia type 1	*Hpd*	Hydrodynamic injection	NHEJ	(Pankowicz et al. 2016)
Hyperammonemia	*OTC*	Adeno-associated virus	HDR	(Yang et al. 2016)
Duchenne muscular dystrophy	*Dmd*	Germline injection	HDR	(Long et al. 2014)
Retinitis pigmentosa	*Rho*	Electroporation	NHEJ	(Bakondi et al. 2016)
Huntington’s disease	*HTT*	Adeno-associated virus	NHEJ	(Yang et al. 2017)
Meesmann’s epithelial corneal dystrophy	*KRT12*	Hydrodynamic injection	NHEJ	(Courtney et al. 2015)

## Delivering Cas9 and gRNA for in vivo gene editing

The practicality of in vitro gene editing using Cas9 is relatively well established (Wang et al. 2017). For gene therapy, both ex vivo and in vivo methods can be used to deliver technologies to cells. Ex vivo delivery involves removing cells or tissues from patients for editing, then engrafting the edited cells back into the patient. However, this review limits the discussion to only in vivo editing. While the potential applications of its in vivo use are apparent, delivery of Cas9 and gRNA into the target cells of the patient is the major obstacle (Wang et al. 2017; Yin et al. 2017; Mout et al. 2017). The required components can be delivered together or separately as either DNA, RNA, or ribonucleoprotein through viral or non-viral approaches. Ideally, components required for gene editing are only delivered to necessary cells in patients, although many current approaches lack the required target specificity. Here, we discuss the features of nucleic acid delivery by either viral or non-viral vectors as well as ribonucleoprotein delivery.

Viral vectors are designed to deliver a payload to the target cells by utilizing the viral infection pathway, while most of the non-essential viral genome is removed from the vector. Integration-deficient lentiviruses, adenoviruses, and adeno-associated viruses are the most popular viral vectors for gene therapy due to their non-integrating nature, eliminating the risks of mutagenesis and tumorigenicity associated with gene insertion (Yin et al. 2017; Lukashev and Zamyatnin 2016; Lundstrom 2018). Among them, adeno-associated viruses are particularly attractive for their low immunogenicity and broad ability to target specific tissues, including liver, brain, skeletal, kidney, retina, lung, and vascular tissue (Mingozzi and High 2011). In comparison, adenoviral vectors suffer from high immunogenicity, while lentiviral vectors normally lack tissue specificity (Yin et al. 2017; Escors and Breckpot 2010). However, the large size of the *SpCas9* gene (4.3 kilobases) poses a challenge to pack into a single adeno-associated virus vector along with the required gRNA (Wu et al. 2010; Senis et al. 2014). One option is to use two vectors, one encoding *Sp*Cas9 and the other encoding the gRNA. The two vectors will be delivered simultaneously, an approach requiring efficient co-transduction of the target cells (Long et al. 2016; Amoasii et al. 2018; Swiech et al. 2015). Alternatively, the smaller *Staphylococcus aureus* Cas9 (3.2 kilobases) can be used, allowing a single vector to encode both the *Sa*Cas9 and gRNA (Ran et al. 2015). However, viral systems can induce long-term transgene expression in humans with a single injection; thus, the potential induction of immunogenicity against Cas9 protein and increase in off-target editing due to sustained endonuclease expression need to be taken into consideration (Yin et al. 2017; Fu et al. 2013).

Non-viral delivery methods, such as hydrodynamic injection and electroporation, have the potential to transfer large genetic payloads with the advantage of a transient expression pattern (Yin et al. 2014b). Hydrodynamic injection is the rapid delivery of a large volume of DNA-containing solution via intravenous injection (Liu et al. 1999). This has been used to deliver components required for Cas9-mediated gene editing in mouse (Yin et al. 2014a; Zhen et al. 2015) and rat models (Bakondi et al. 2016). However, as a large injection volume (about 10% of the animal’s body weight) is required, hydrodynamic injection is unlikely to be suitable for human applications. Alternatively, electroporation, the stimulation of cells via electrical pulse, can also facilitate cellular uptake of foreign components specific to particular tissue, and its use has also been demonstrated in animal models (Bakondi et al. 2016; Xu et al. 2016). However, due to the large amount of cell death induced in the treatment area by this method, electroporation has yet to be employed in human clinical trials. In addition, neither hydrodynamic injection nor electroporation shows cell specificity.

Ribonucleoprotein, composed of Cas9 and gRNA, can be directly delivered into cells for genetic modification. In comparison to the delivery of DNA or RNA to generate ribonucleoprotein in vivo, delivery of ribonucleoprotein is appealing as the molecules are immediately active, resulting in rapid editing (Kim et al. 2014). This method is also recognized for its limited half-life, which reduces potential off-target editing (Liang et al. 2015). A major challenge of ribonucleoprotein delivery is packaging Cas9 protein with gRNA. Cationic lipids such as RNAiMAX (Zuris et al. 2015) have enabled gene editing of up to 20% in mouse models and have entered clinical testing for other gene therapies (Fitzgerald et al. 2014; Coelho et al. 2013). Gold nanoparticles have also been used to deliver the ribonucleoprotein in mouse models (Lee et al. 2017a). The nanoparticles were complexed with donor DNA, Cas9 RNP, and PAsp(DET). PAsp(DET) is a polymer that induces both endocytosis and later endosomal disruption to release CRISPR components into the cytosol. Although target-specific delivery by these means are yet limited to local injections, recent development of receptor-mediated ribonucleoprotein delivery has shown cell specificity in vitro (Rouet et al. 2018), indicating the possibility of targeted ribonucleoprotein delivery.

Many factors need to be considered when selecting the delivery vector. An ideal delivery method should have high specificity to the diseased cells and tissues, be non-immunogenic and non-toxic to the host, and enable transient Cas9 activity to minimize potential off-target editing. Although none of the currently available delivery methods fulfill all these criteria (Table 2), it is possible to use a switchable Cas9 variant to control tissue specificity and the duration of Cas9 activity.

**Table 2:** Comparison of different methods to delivery Cas9 and gRNA

	Tissue tropism	Form of delivery	Loading capacity	Duration of Cas9 activity	Other considerations	References
Integration-deficient lentiviruses	*Maybe*	DNA	8 kb	*Weeks to months*		(Yin et al. 2017; Escors and Breckpot 2010; Milone and O’Doherty 2018)
Adenoviruses	Yes	DNA	8–36 kb	*Weeks to months*	*High immunogenicity*	(Lee et al. 2017b)
Adeno-associated viruses	Yes	DNA	*5 kb*	*Years*		(Mingozzi and High 2011)
Hydrodynamic injection	*No*	DNA	Unlimited	Days	*High injection volume required*	(Yin et al. 2014a; Zhen et al. 2015; Bakondi et al. 2016)
Electroporation	*Limited*	DNA	Unlimited	Days	*High level of cell death*	(Bakondi et al. 2016; Xu et al. 2016)
Cationic lipids	*No*	Protein	Unlimited	< 48 h		(Zuris et al. 2015)
Gold nanoparticles	*Maybe*	Protein	Unlimited	< 48 h		(Lee et al. 2017a)
Receptor-mediated	Yes	Protein	Unlimited	< 48 h	*Endosomal escape difficult*	(Rouet et al. 2018; Guan and Rosenecker 2017)

Limitation(s) and main consideration(s) of the delivery methods are shown in italic

## Switchable Cas9

Cas9 variants that can be regulated by an external stimulus are of great interest to therapeutic development, as they allow an extra level of spatial and temporal control over gene editing (Gangopadhyay et al. 2019; Nihongaki et al. 2018; Richter et al. 2017; Zhou and Deiters 2016). Improved spatial resolution limits activity to specific cells and tissues, if this is not already conferred by the delivery vector, whereas control over temporal resolution can minimize off-target editing by confining the duration of active Cas9 in cells (Hu et al. 2018; Yin et al. 2014a). To date, different switchable Cas9 variants have been developed (Gangopadhyay et al. 2019; Nihongaki et al. 2018; Richter et al. 2017; Zhou and Deiters 2016), and their activity can be controlled by temperature (Richter et al. 2016; Jiang et al. 2019), light (Nihongaki et al. 2015; Hemphill et al. 2015; Richter et al. 2016; Jain et al. 2016; Zhou et al. 2018), or small molecules (Suzuki et al. 2018; Zetsche et al. 2015; Davis et al. 2015; Oakes et al. 2016; Liu et al. 2016; Nguyen et al. 2016; Tang et al. 2017; Senturk et al. 2017; Rose et al. 2017). These variants work well in vitro; however, their applicability for controlling in vivo gene editing is likely to greatly depend on the nature of the stimuli.

Temperature-sensitive Cas9 variants (Richter et al. 2016; Jiang et al. 2019) are unlikely to be suitable for controlling gene editing inside a human body. The body temperature of humans is normally maintained at 37 °C with minimal fluctuation. It will therefore be challenging to maintain a target tissue at an alternative temperature for an extensive period for gene editing to take place. This holds even for a Cas9 variant which is active at 29 °C instead of 37 °C (Richter et al. 2016). Conversely, light is a unique stimulus that offers superior spatial control to subcellular levels. However, the major drawback of using light to modulate Cas9 function is the limited tissue penetrability (Ash et al. 2017), making this approach unfeasible when targeting internal organs. To date, light-responsive Cas9 variants can be controlled by either 365 nm (Hemphill et al. 2015; Jain et al. 2016), 470 nm (Nihongaki et al. 2015; Richter et al. 2016), or 500 nm (Zhou et al. 2018) wavelength light, which can penetrate tissues at about 700, 1600, or 2500 μm depth respectively (Ash et al. 2017). Therefore, their uses may be limited to skin diseases, such as epidermolysis bullosa simplex (Lewin et al. 2005), as the depth of the epidermis is within 130 μm (Sandby-Moller et al. 2003).

Small molecules arguably hold the greatest potential for controlled in vivo activation of Cas9. High temporal control is achieved by the time of administration and dosage of the small molecule. Although high spatial resolution with this approach can only be achieved by local administration of the modulator molecules (Han et al. 2017), small molecules can theoretically reach any tissues within a human body, unlike regulation by temperature or light. Ideally, a small-molecule Cas9 modulator should have no effects on other proteins or biomolecules, preventing disturbance of other cellular processes. Unfortunately, most switchable Cas9 variants developed to date are responsive to drug molecules, such as antibiotic rapamycin (Zetsche et al. 2015), estrogen receptor modulator 4-hydroxytamoxifen (Davis et al. 2015; Oakes et al. 2016; Liu et al. 2016; Nguyen et al. 2016), Bcl-XL inhibitor A-385358 (Rose et al. 2017), and respiratory drug theophylline (Tang et al. 2017). Nevertheless, there are two approaches to control Cas9 activity by non-drug molecules, Shield-1 (Senturk et al. 2017), and Lys(Boc) (Suzuki et al. 2018).

**Fig. 5:**
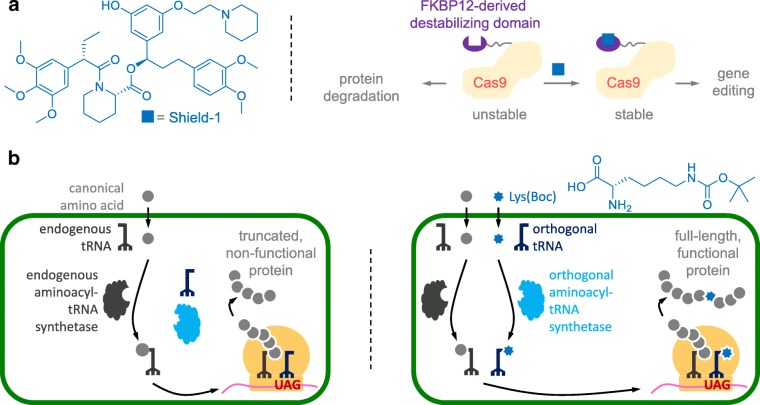
Regulation of *Sp*Cas9 by non-drug molecules. **a** Stability of the fusion protein containing *Sp*Cas9 and a FKBP12-derived destabilizing domain can be regulated by Shield-1 so that in the absence of Shield-1, all fusion proteins are rapidly degraded. **b** Genetic code expansion for site-specific non-canonical amino acid incorporation is used to control the production of full-length, functional *Sp*Cas9

Shield-1, a ligand that binds to and stabilizes an FKBP12-derived destabilizing protein domain, is highly cell permeable, and has no in vivo toxicity (Banaszynski et al. 2008). By fusing the destabilizing domain to *Sp*Cas9, the resultant destabilized Cas9 protein variant was not detectable in the absence of Shield-1 (Fig. 5a). Upon addition of Shield-1 into the culture media, Cas9 protein was detected within 2 h, whereas subsequent removal of Shield-1 from the culture media led to depletion of Cas9 protein within 12 h (Senturk et al. 2017).

Lys(Boc) is an economic, non-canonical amino acid that does not show any observable toxicity to cell lines and embryos (Suzuki et al. 2018). It can be site-specifically incorporated into a protein of interest in mammalian cells using genetic code expansion. In mammalian cells, proteins composed of 20 canonical amino acids are produced by ribosome, which employs aminoacyl-tRNAs to decode the information on mRNA to generate the corresponding protein. To expand the genetic code, an orthogonal aminoacyl-tRNA synthetase/tRNA pair is introduced into the cell. The orthogonal synthetase specifically acylates the orthogonal tRNA with a designated non-canonical amino acid, such as Lys(Boc), to generate the required aminoacylated tRNA (Nödling et al. 2019). Here, orthogonality means that the orthogonal synthetase does not use any of 20 canonical amino acids nor any of the endogenous tRNA as substrate, and the non-canonical amino acid is *not* a substrate of any of the endogenous synthetases. The orthogonal tRNA recognizes a blank codon on the mRNA to direct incorporation of the non-canonical amino acid into the target protein. Amber stop codon (UAG) is usually used as the blank codon due to its rarity among the three stop codons in most organisms. Pyrrolysyl-tRNA synthetase/tRNA pair from the archaea *Methanosarcina* species is an orthogonal pair in mammalian cells and can direct Lys(Boc) incorporation in response to an amber codon (Nödling et al. 2019)**.**

An *SpCas9* gene harboring a centrally located amber codon alongside a pyrrolysyl-tRNA synthetase/tRNA pair has been successfully used to control gene editing in mouse embryos (Suzuki et al. 2018). In the absence of Lys(Boc), truncated and non-functional *Sp*Cas9 is obtained (Fig. 5b), whereas supplementation of the non-canonical amino acid led to production of full-length and functional *Sp*Cas9 protein. This approach enables heritable Cas9-mediated mammalian genome editing that is acutely controlled by the economic and readily available lysine derivative (Suzuki et al. 2018). However, amber suppression may interfere with translation of endogenous genes ending with the amber stop codon. In addition, the system requires multiple components to be delivered to the cell and, as previously discussed, vectors capable of delivering a large cargo are limited.

Currently, duration of Cas9 activity in vivo depends on the delivery methods as described in Table 2. Switchable Cas9 variants can offer a solution to long-term transgene expression and subsequent off-target editing associated with viral delivery vectors (Yin et al. 2017; Fu et al. 2013). The great temporal control of switchable Cas9 variants enables accurate regulation of genetic modification, circumventing the concerns over extended activity timeframes associated with delivery of Cas9 DNA. Cas9 variants regulated by Shield-1 (Senturk et al. 2017) and Lys(Boc) (Suzuki et al. 2018) can be switched on and off easily by the presence or absence of the required small molecule, enabling delicate regulation of Cas9 activity and greater tissue specificity through local administration of the molecule. Despite the advantages offered by switchable Cas9 variants, their uses in disease animal models and therapeutic applications are still very limited. Considerable further development of these switchable variants is therefore needed before they can be applied to the clinical setting.

## Conclusions

Progress in gene editing techniques have improved rapidly in recent years and benefited by the discovery of CRISPR/Cas9. The versatile and highly specific gene editing achieved by Cas9 is so far the most promising approach for correction of genetic diseases. The potential for many genetic diseases to be resolved in a permanent and heritable fashion by this technology is clear and may be achievable in the near future especially for diseases that could benefit from repair by the NHEJ mechanism. However, significant improvement in HDR efficiency is required before the power of this repair mechanism can be fully exploited for the therapeutic applications. Regardless of the repair mechanism, a major hindrance of in vivo gene editing thus far has been the lack of a suitable delivery vector with cell specificity, while providing transient Cas9 delivery and low immunogenicity.

Fortunately, switchable Cas9 variants offer solutions to some of the obstacles. Specifically, the great temporal control of switchable Cas9 variants enables rapid and accurate regulation of genetic modification, circumventing the extended activity timeframe associated with some means of Cas9 delivery, whereas the spatial control of switchable Cas9 variants could provide target specificity if not conferred by delivery vectors. However, detailed in vivo investigations of switchable Cas9 variants are required to translate their use into clinical applications. Unfortunately, protein scientists working on Cas9 engineering often lack in vivo expertise. Thus, it will be necessary that scientists working on tool and therapeutic development closely collaborate, so the true potential of Cas9-mediated gene therapy can be transformed into clinical settings.
